# Effects of the γ-secretase inhibitor semagacestat on hippocampal neuronal network oscillation

**DOI:** 10.3389/fphar.2013.00072

**Published:** 2013-06-14

**Authors:** Mihály Hajós, Elena Morozova, Chester Siok, Kevin Atchison, Charles E. Nolan, David Riddell, Tamás Kiss, Eva Hajós-Korcsok

**Affiliations:** ^1^Laboratory of Translational Neuropharmacology, Section of Comparative Medicine, Yale University School of MedicineNew Haven, CT, USA; ^2^Pfizer Inc.Groton, CT, USA

**Keywords:** γ-secretase inhibitors, LY450139, theta, hippocampus, electroencephalography, Alzheimer’s disease

## Abstract

Neurological and psychiatric disorders are frequently associated with disruption of various cognitive functions, but development of effective drug treatments for these conditions has proven challenging. One of the main obstacles is the poor predictive validity of our preclinical animal models. In the present study the effects of the γ-secretase inhibitor semagacestat was evaluated in preclinical *in vivo* electrophysiological models. Recently disclosed Phase III findings on semagacestat indicated that Alzheimer’s disease (AD) patients on this drug showed significantly worsened cognitive function compared to those treated with placebo. Since previous studies have shown that drugs impairing cognitive function (including scopolamine, NMDA (*N*-methyl-D-aspartate) receptor antagonists, and nociceptin receptor agonists) disrupt or decrease power of elicited theta oscillation in the hippocampus, we tested the effects of acute and sub-chronic administration of semagacestat in this assay. Field potentials were recorded across the hippocampal formation with NeuroNexus multi-site silicon probes in urethane anesthetized male C57BL/6 mice; hippocampal CA1 theta oscillation was elicited by electrical stimulation of the brainstem nucleus pontis oralis. Sub-chronic administration of semagacestat twice daily over 12 days at a dose known to reduce beta-amyloid peptide (Aβ) level [100 mg/kg, p.o. (per oral)] diminished power of elicited hippocampal theta oscillation. Acute, subcutaneous administration of semagacestat (100 mg/kg) produced a similar effect on hippocampal activity. We propose that the disruptive effect of semagacestat on hippocampal function could be one of the contributing mechanisms to its worsening of cognition in patients with AD. As it has been expected, both acute and sub-chronic administrations of semagacestat significantly decreased Aβ40 and Aβ42 levels but the current findings do not reveal the mode of action of semagacestat in disrupting hippocampal oscillignificantly reduced braination.

## INTRODUCTION

Neurological and psychiatric disorders are frequently associated with disruption of various cognitive functions, but development of effective drug treatments for these conditions has proven challenging. One of the main obstacles is the poor predictive validity of our preclinical animal models; drugs showing improved cognitive function in behavioral rodent models often fail in the clinics, in part due to the shortcomings of modeling disease pathology in experimental animals.

Using *in vivo* electrophysiological assays, previous studies have shown that drugs impairing cognitive function, such as scopolamine, NMDA (*N*-methyl-D-aspartate) receptor antagonists and nociceptin receptor agonists disrupt or decrease power of elicited theta oscillation in the hippocampus of anesthetized rats (Kinney et al., 1999; Siok et al., 2006; Li et al., 2007; McNaughton et al., 2007; Hajós et al., 2009; Kittelberger et al., 2012) or mice (Guadagna et al., 2012; Kiss et al., 2013). Furthermore, in transgenic mice capturing some pathology of a neurological or psychiatric disease and showing cognitive impairment power of elicited hippocampal theta is significantly reduced, including the beta-amyloid peptide (Aβ) overproducing amyloid precursor protein (APP)/PS1transgenic mice, tau-transgenic mice and NMDA receptor hypomorphic mice (Scott et al., 2011, 2012; Kiss et al., 2013). In contrast, those few drugs which improve cognitive function in patients, including acetylcholine esterase inhibitors and memantine increase the power of elicited theta oscillation in this assay (Kinney et al., 1999; Guadagna et al., 2012).

In the present study the effects of the γ-secretase inhibitor (GSI) semagacestat has been evaluated in this preclinical hippocampal theta model. One of the distinguishing features of Alzheimer’s disease (AD) pathology is deposition of Aβ containing senile plaques in the brains of patients. According to the amyloid hypothesis, increased production or decreased clearance and degradation of Aβ initiates a molecular cascade ultimately leading to neurodegeneration and the clinical manifestation of cognitive decline and senile dementia. The generation of Aβ requires sequential cleavage of the type I integral membrane APP by β then γ-secretase (Imbimbo and Giardina, 2011). Semagacestat, by inhibiting γ-secretase reduces level of Aβ in Alzheimer’s patients (Bateman et al., 2009; Mancuso et al., 2011), therefore it was predicted that it could either improve cognitive function and/or slow down disease progression. However, recently disclosed Phase III findings on semagacestat indicated that AD patients on this drug showed significantly worsened cognitive function to those treated with placebo (Schor, 2011). In our studies, we evaluated the effects of semagacestat on hippocampal theta oscillation in normal mice (C57/BL/6), both after sub-chronic and acute administrations. For sub-chronic treatment of semagacestat, mice received oral drug treatment at 100 mg/kg dose, twice daily over 12 days; electrophysiological recordings were carried out 3 h after the last drug administration. Acute effects of semagacestat were also evaluated after subcutaneous administration of semagacestat (100 mg/kg). After completion of experiments brain samples were collected for Aβ level measurements; drug exposure levels were determined from both plasma and brain samples.

## MATERIALS AND METHODS

### SURGICAL PROCEDURES AND ELECTROPHYSIOLOGICAL RECORDINGS

Male C57BL/6 mice were anesthetized with 1.5–1.7 g/kg urethane intraperitoneally, under an approved animal use protocol and in compliance with the Animal Welfare Act Regulations [9CFR (Code of Federal Regulations) parts 1, 2, and 3] and with the Guide for the Care and Use of Laboratory Animals, National Institutes of Health guidelines. The animals were placed in a Kopf stereotaxic frame on a temperature regulated heating pad (Harvard Apparatus) set to maintain body temperature at 37–38 °C.

### STIMULATION-EVOKED HIPPOCAMPAL THETA OSCILLATION

**FIGURE 1:**
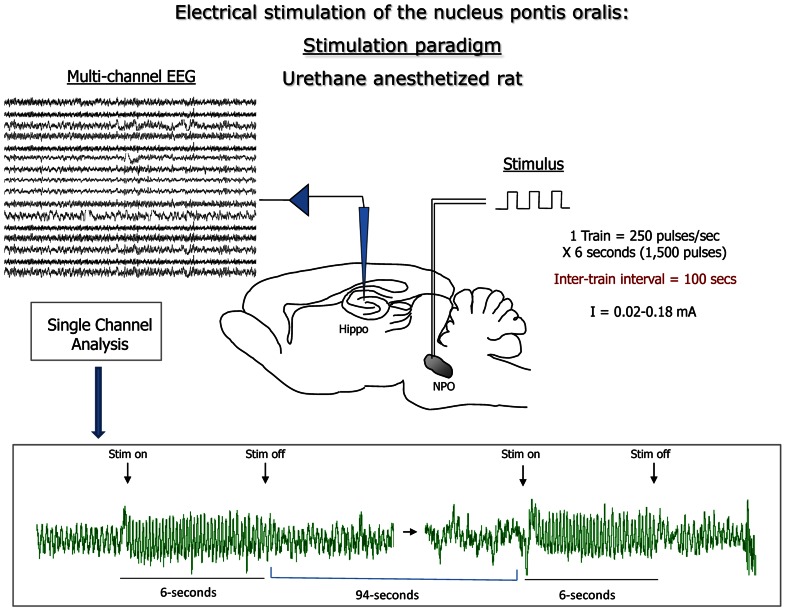
**Summary of the protocol used in the present study: electrical stimulation of the brainstem nucleus pontis oralis (nPO; with increasing current intensity between first and second simulation; indicated as Stim on and Stim off) elicited rhythmic oscillation of hippocampal local field potential (LFP) in theta frequency; recordings were carried out across the hippocampal formation; LFP from the hippocampus CA1 region was analyzed in the present study.** Following fast Fourier transformation (FFT) of the LFP frequency of the highest power component (Peak frequency), and total power in the theta frequency band (3–12 Hz) were recorded for each stimulation period.

A 16-site silicon recording electrode (A1x16-10mm-100-177-T15 NeuroNexus Technologies, Inc, Ann Arbor, MI, USA) was acutely implanted to span the hippocampal formation, placed 2.0 mm posterior from and 1.5 mm lateral to bregma and the tip slowly lowered 1.9 mm from the cortical surface (Paxinos and Franklin, 2001). As a first step, population spikes in the dentate gyrus were recorded in response to stimulation of perforant path (co-ordinates of stimulating electrode:: 0.5 mm anterior to and 2.5 mm lateral to lambda and 1.4–1.9 mm from cortical surface) using a concentric bipolar electrode (NE-100X, Rhodes Medical Instruments, Woodland Hills, CA, USA) in order to position recording electrodes consistently in all mice, as we reported previously (Scott et al., 2012). Subsequently, the same stimulating electrode was positioned to the nucleus pontis oralis (nPO), 4.0 mm posterior and 1.2 mm lateral from bregma and 3.3 mm ventral from cortical surface. Stimulation consisted of a train of 0.3 ms square pulses delivered over 6 s at 250 Hz, repeated every 100 s (**Figure 1**). Stimulus intensities were either held constant for time course experiments or increased in a step-wise fashion (see below). During each experiment, spontaneous and stimulation-induced local field potentials were continuously monitored, data was digitized at 1 kHz using the A-M System (Carlsborg, WA, USA) and Spike2 software package (Cambridge Electronic Design, Cambridge, UK). Among the 4 out of 16 channels located in the CA1 region one channel was selected in this study for further analysis and statistics.

Comparing theta activity in mice sub-chronically treated with vehicle or semagacestat stimulus-response curves were obtained using increasing stimulus intensities of 0.00–0.20 mA in 0.02 mA increments, 3 h after last oral drug administration. Input–output curves were generated by repeating this stimulation paradigm five times per animal; the last three measurements were averaged for analysis. Acute effects of semagacestat were tested following determination of stimulus-response relationship for both peak theta frequency and total theta power for each mouse. Stimulating current necessary to induce hippocampal theta oscillation between 5 and 8 Hz with an absolute power between 60 and 80% of the maximal response was used for time course experiments as described in detail earlier (Siok et al., 2006).

### ELECTROPHYSIOLOGICAL DATA ANALYSIS AND STATISTICS

For nPO stimulation-evoked hippocampal theta oscillation Fast Fourier transformation (FFT) was performed on each 6 s stimulation period, using 1–5.096 s (0.25 Hz frequency resolution) following stimulation onset, to avoid inclusion of stimulation artifact in analysis. Peak frequency and total power in the theta band (3–12 Hz) were determined. For visual assessment of the composition of hippocampal oscillations short-time FFTs were calculated. Frequencies between 0.25 and 10 Hz were evaluated by a 0.05 Hz frequency step. To decrease granularity of the resulting high-resolution figures data was smoothed using a two-dimensional square function of 1 s × 0.5 Hz size. These calculations were performed in Matlab (R2009a, MathWorks, Inc, Natick, MA, USA). For statistical evaluation of data either GraphPad Prism (GraphPad Software, Inc, La Jolla, CA, USA) or the Matlab Statistics Toolbox (v7.1, R2009a, MathWorks, Inc, Natick, MA, USA) was used.

### BIOCHEMISTRY

#### Homogenization of brain tissue

Frozen C57BL/6 brain tissue was homogenized in 10 volumes of 5 M guanidine-HCl, pH 8 (Sigma-Aldrich, St Louis, MO, USA) using a Tissue Lyser II (Qiagen, Germantown, MD, USA) and incubated for 3 h at room temperature. The guanidine homogenate was centrifuged at 116,000 × *g* for 1 h, 4°C. The resulting supernatant was applied to 60 mg HLB 96-well plates (Waters, Milford, MA, USA) and concentrated as described previously (Lanz and Schachter, 2006). The resulting lyophilized pellets were stored at -80°C prior to resuspension in blocking buffer for analysis by the DELFIA Aβ ELISA.

### DELFIA AβELISA

Resuspended brain tissue lysates were incubated in 384-well black plates prepared as follows. 384-well black plates (VWR, Bridgeport, NJ, USA) were coated overnight at 4°C with a C-terminal-specific anti-Aβ40 (4 μg/ml), and an anti-Aβ42 (10 μg/ml; Rinat, South San Francisco, CA, USA), diluted in 0.1 M sodium bicarbonate, pH 8.2. The next day plates were washed and then blocked for 2–4 h at room temperature with 1% bovine serum albumin in phosphate buffered saline with 0.05% Tween 20 (Sigma-Aldrich, St. Louis, MO, USA). Standard curves were prepared from stock solutions of species-specific Aβ peptides (Bachem Biosciences, King of Prussia, PA, USA) in blocking buffer. Standards and samples were incubated on the coated and blocked 384-well plates overnight at 4°C. Plates were washed and a secondary biotinylated 4G8 (0.2 μg/ml; Covance, Dedham, MA, USA) was incubated for 2 h at room temperature. The signal was amplified by incubation with europium-conjugated streptavidin for 1 h at room temperature followed by incubation with DELFIA enhancement solution at room temperature for 20 min in the dark. Plates were read on an EnVisionMultilabel plate reader (europium-Delfia reagents and equipment from PerkinElmer Life Sciences, Boston, MA, USA). Standard values were fit to a fourth-order polynomial curve, and sample values were extrapolated using GraphPad Prism 5.02 (La Jolla, CA, USA).

### DRUGS

For acute administration semagacestat (LY450139, synthesized at Pfizer, Inc., Groton, CT, USA) 100 mg/kg or vehicle (Phosal/Tween, 12/88) were injected subcutaneous (s.c.) after 30 min of stable baseline recording. For sub-chronic administration semagacestat 100 mg/kg or vehicle (20%PEG/20%Solutol) were administered per oral (p.o.) twice daily with last drug application done 3 h before the start of recording.

### MEASUREMENT OF DRUG LEVELS IN THE BRAIN AND PLASMA

Semagacestat exposure was determined after completion of the electrophysiological measurements, 5 h following the last oral administration of semagacestat in the sub-chronic study, or 3 h after its subcutaneous injection. Concentration of semagacestat was determined using liquid chromatography-tandem mass spectrometry following the method reported previously (Lanz et al., 2006).

## RESULTS

### REDUCED POWER OF ELICITED HIPPOCAMPAL THETA OSCILLATION IN RATS SUB-CHRONICALLY TREATED WITH SEMAGACESTAT

**FIGURE 2:**
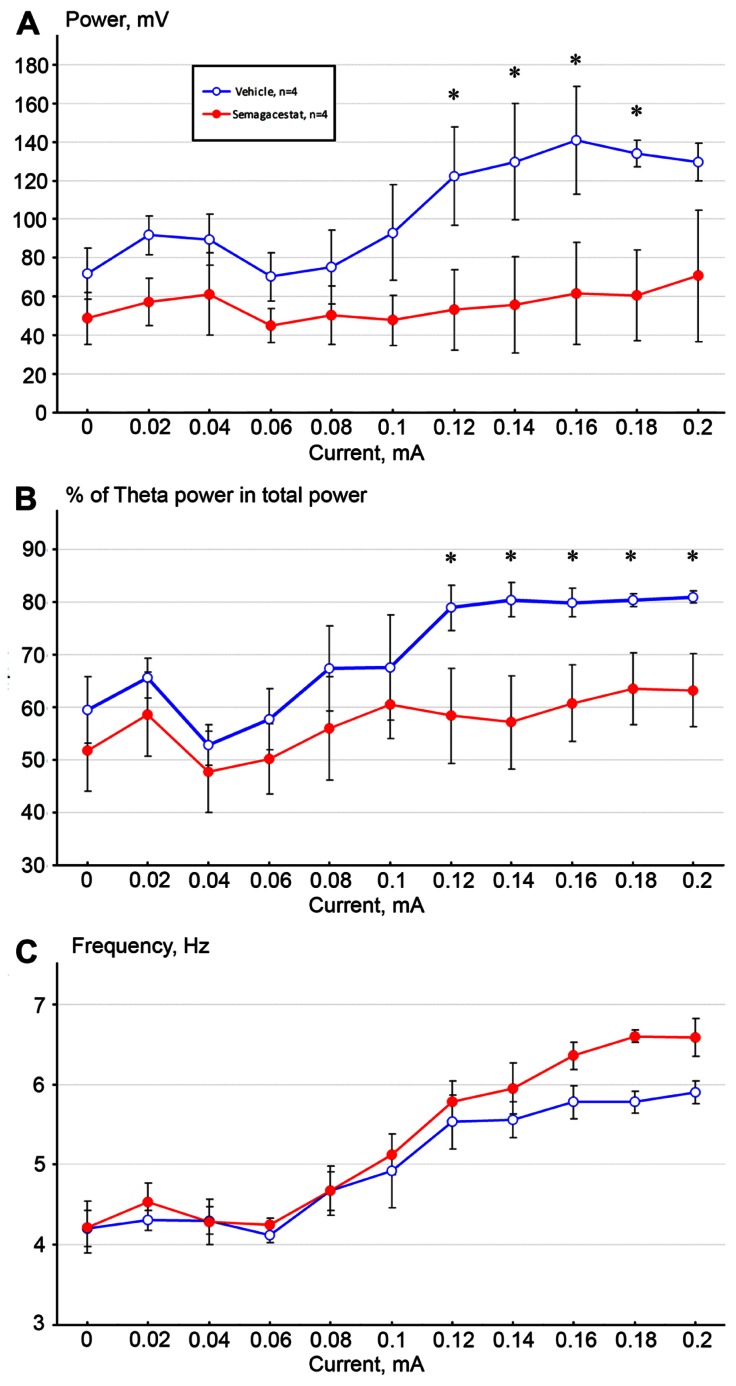
Sub-chronic administration of semagacestat (twice daily 100 mg/kg, p. o. over 12 days, *n* = 4, RED symbols) significantly attenuated absolute (A) and relative (B) power of hippocampal theta oscillation elicited by stimulation of the nucleus pontis oralis with increasing currents (0–0.2 mA) compared to vehicle-treated control C57BL/6 mice (*n* = 4; BLUE symbols; *p < 0.05). In contrast, no significant difference in peak frequency in elicited theta activity was noticed (C).

Electrical stimulation of the nPO elicited highly regular hippocampal oscillations whose frequency and amplitude increased proportionally to the stimulus intensity in vehicle-treated C57BL/6 mice (twice daily 100 mg/kg, p.o. over 12 days, *n* = 4), as shown previously in both anesthetized rats and mice (Kinney et al., 1999; Siok et al., 2006; McNaughton et al., 2007; Scott et al., 2012; Kiss et al., 2013). Sub-chronic administration of semagacestat (*n* = 4) significantly attenuated power of hippocampal theta oscillation across the entire theta frequency range (*p* < 0.05) as measured 3 h following the last orally administered dose of semagacestat. Comparing baseline level of absolute theta power, increasing stimulation currents failed to elicit higher theta power (**Figure 2A**), and relative theta power stayed at the same range as well (**Figure 2B**). In contrast, increasing stimulating current induced the same increase in theta frequency between vehicle- and semagacestat-treated mice, in fact there was a trend to augmented theta frequency in semagacestat-treated mice at higher stimulation currents/higher frequency range (**Figure 2C**).

### ACUTE ADMINISTRATION OF SEMAGACESTAT REDUCES POWER OF ELICITED HIPPOCAMPAL THETA OSCILLATION

**FIGURE 3:**
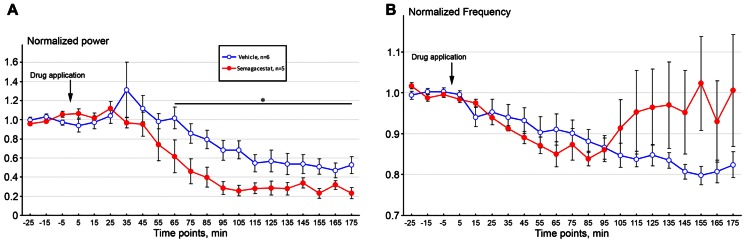
Acute administration of semagacestat (100 mg/kg, s. c, *n* = 5, RED symbols) significantly (*p < 0.05) attenuated power of elicited hippocampal theta oscillation (A) compared to vehicle-treated control C57BL/6 mice (*n* = 6, BLUE symbols) as followed in time. No significant difference in peak frequency in elicited theta activity was noticed **(B)**.

**FIGURE 4:**
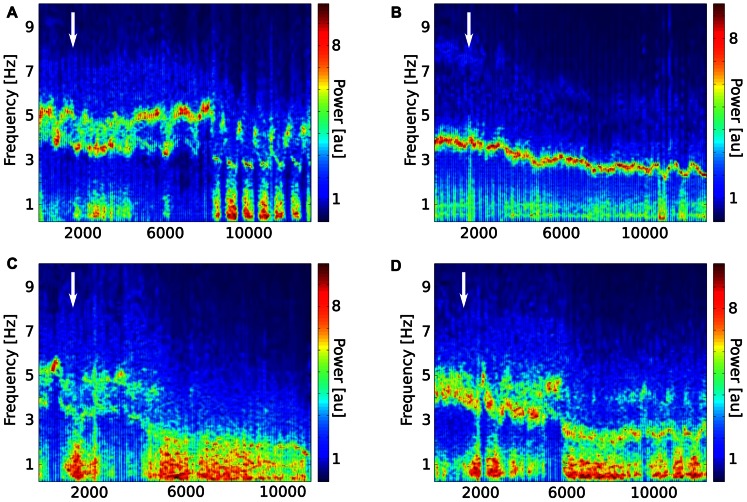
**Time-frequency decomposition of representative hippocampal recordings from two mice treated with vehicle (A,B)or semagacestat (100 mg/kg; C,D) approximately 30 min after start of recordings as indicated by white arrows.** Note the strong and persistent low frequency (<2 Hz) component of the ongoing oscillation pattern in the semagacestat-treated animals, which unlike in the case of vehicle-treated animals does not give place to the regular 4–5 Hz theta rhythm during stimulation periods.

Effects of acutely administered semagacestat were also evaluated on stimulation-induced hippocampal theta oscillation. In these experiments, stimulating current was determined in each individual mouse by establishing a stimulus-response relationship; and current inducing theta oscillation between 5 and 8 Hz frequency with an absolute power between 60 and 80% of the maximal response was selected. For inter-animal comparisons, total theta power during nPO stimulation was normalized for each mouse to the average power measured prior to drug or vehicle administration. Following 30 min baseline recordings semagacestat (100 mg/kg, s.c, *n* = 5) or it vehicle (*n* = 6) was given and stimulation-induced theta oscillation followed up to 3 h. As it has been expected, power of stimulation-induced theta gradually decreased through the recording period, however, semagacestat-treated mice showed a significantly greater reduction in theta power then vehicle-treated mice(*p < 0.05; **Figure 3**). Time-frequency decomposition of representative hippocampal recordings also indicated that power of spontaneous hippocampal field potential shifted to lower (<2Hz) frequencies, and nPO stimulation did not elicit the regular 4–5 Hz theta rhythm, as it is demonstrated on **Figure 4**. No significant difference in peak frequency in elicited theta activity was noticed between drug and vehicle-treated mice, although a clear trend for an increase in frequency in semigacestat-treated mice was noticed at approximately 90 min after drug administration (**Figure 3B**).

### BOTH SUB-CHRONIC AND ACUTE ADMINISTRATIONS OF SEMAGACESTAT SIGNIFICANTLY REDUCED BRAIN Aβ SPECIES

**FIGURE 5:**
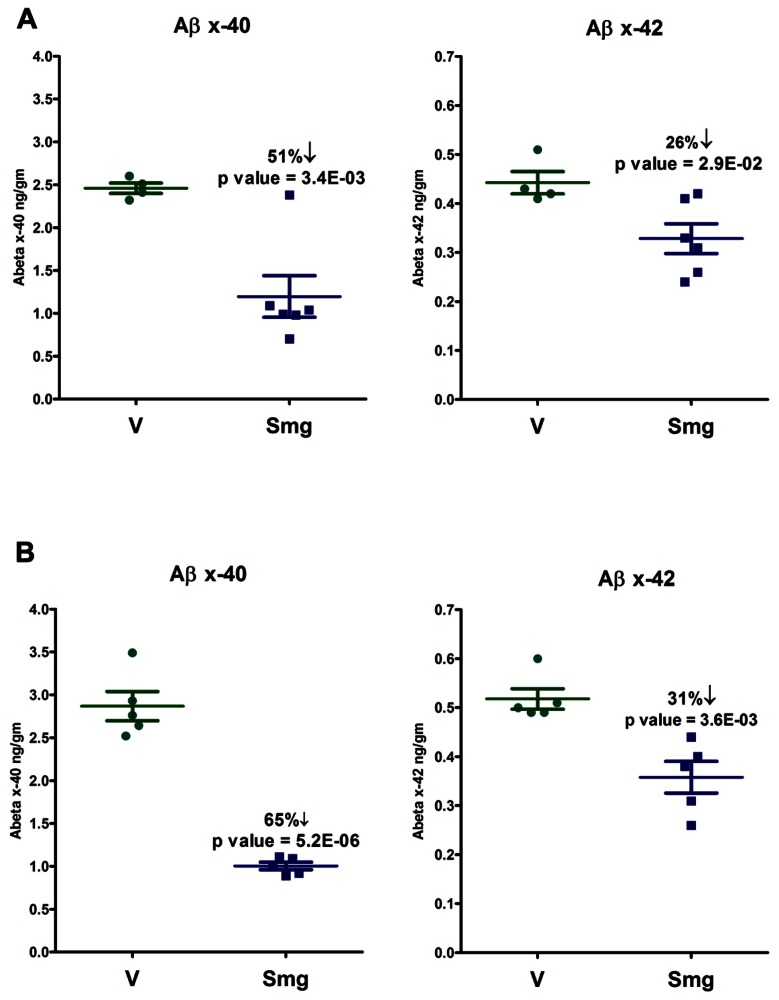
**Both sub-chronic treatment (A, twice daily, 100 mg/kg, p. o. over 12 days) and acute treatment (B, 100 mg/kg, s.c.) with semagacestat (Smg) significantly reduced brain Aβ levels compared to vehicle-treated controls**.

Beta-amyloid peptide levels, extracted from C57BL/6 mouse brain homogenates acutely or sub-chronically treated with semagacestat, were measured using DELFIA TRF technology. The resulting Aβ lowering in the sub-chronically dosed mice were observed to be significant at 51% for Aβ40 and 26% for Aβ42 comparing to vehicle-treated mice, approximately 4–5 h after last oral drug administration and on completion of the electrophysiological recording (**Figure 5A**). Acutely treated mice (100 mg/kg, s.c.) had an even greater reduction in Aβspecies after completion of the electrophysiological recordings, and 3 h after drug administration, showing a high level statistical significance, as a 65% lowering of Aβ40 and a 31% reduction of Aβ42. Note the greater lowering of Aβ40 with respect to Aβ42 in both the acute and sub-chronically treated mice. This reflects an apparent GSI-induced increase in the ratio of Aβ42 to Aβ40 (**Figure 5B**).

### PLASMA AND BRAIN EXPOSURE OF SEMAGACESTAT

Completion of the electrophysiological recording blood and brain samples were taken for determining levels of semagacestat. Both sub-chronic and acute s.c. administration resulted in the expected range of drug exposure in mice. Unbound brain compound concentrations were 0.14 ± + 0.03 and 4.9 ± + 0.5 μM; unbound plasma compound concentrations were 1.6 ± + 0.04 and 51 ± + 10 μM following sub-chronic and acute administration of semagacestat, respectively. Blood/plasma ratio of 0.12 is similar to previously reported value obtained in guinea-pigs (Lanz et al., 2006).

## DISCUSSION

Both sub-chronic and acute treatment of anesthetized mice with the GSI semagacestat significantly impacted stimulation-induced hippocampal theta activity, primarily reducing power of theta oscillation. This effect is characteristic of drugs which are known to disrupt cognitive function in humans or animal models. Although both sub-chronic and acute semagacestat treatments significantly reduced brain Aβ40 and Aβ42 levels, the exact mechanism(s) underlying the currently described neurophysiological effect of semagacestat is unknown at the present.

In line with previous studies, high frequency stimulation of the nPO elicited hippocampal theta oscillation, characterized by a current-dependent increase in both theta frequency and theta power in vehicle-treated mice (Guadagna et al., 2012; Scott et al., 2012; Kiss et al., 2013). Mice treated sub-chronically with semagacestat showed a similar current-dependent increase in theta frequency to those mice treated with vehicle; however, the power of theta oscillation was significantly reduced. Comparing either absolute or relative theta power between vehicle- and semagacestat-treated mice, significant differences were found over the entire theta frequency range. In fact, current-dependent increase in theta power was almost absent in animal pretreated with semagacestat. Furthermore, single administration of semagacestat at a dose (100 m/kg, sc) known to acutely reduce brain Aβ peptides (Lu et al., 2012) resulted in a significantly greater reduction in theta power than acute administration of its vehicle. Interestingly, there was a tendency for increasing theta frequency following either sub-chronic or acute administration of semagacestat, although these differences did not reach significant levels. Since a reduction in elicited theta power has been demonstrated by each and every drug known to impair cognition, regardless of their mode of action (for review see McNaughton et al., 2007), we propose that the presently observed effect underlies, at least in part, the observed worsening of clinical measures of cognition and the ability to perform activities of daily living of Alzheimer’s patients treated with semagacestat (Schor, 2011). In fact, semagacestat has been reported to impaired normal cognition in wild-type mice and young Aβ overproducing Tg2576 mice measured in Y-maze task (Mitani et al., 2012). As power of theta oscillation reflects synchronous activity among hippocampal pyramidal neurons, it is presumed that drugs reducing power of theta oscillation disrupt fine tuning of pyramidal neurons and subsequently impairs cognitive function. For example, it has been shown that cannabinoid-1 receptors agonists disrupt temporal coordination of cell assemblies in the hippocampus, which is reflected in disrupted spontaneous or stimulation-induced theta oscillation and leads to memory deficits (Robbe et al., 2006; Hajós et al., 2008; Hajós and Siok, unpublished observations).

As it has been predicted, both sub-chronic and acute administration of semagacestat reduced Aβ species in a fashion known to be characteristic of GSIs (Bateman et al., 2009; Lu et al., 2012). However, it is unknown at the moment if Aβ reduction contributes to the diminished hippocampal theta oscillation. Since recent findings indicate a physiological role of Aβ peptides in synaptic neurotransmission underlying learning and memory (Pearson and Peers, 2006; Morley et al., 2010), this possibility cannot be ruled out. Although microinjection of Aβ into the hippocampus impairs behavioral performance and the associated hippocampal theta oscillation (Villette et al., 2010), and elicited hippocampal theta oscillation is age-dependently disrupted in Aβ overproducing APP/PS1 mice (Scott et al., 2012) indicating a synaptic failure (Selkoe, 2002), recent findings also demonstrate a more complex mechanism of Aβ action on neuronal excitability (Busche et al., 2012; Mucke and Selkoe, 2012; Verret et al., 2012). Testing additional GSIs in this electrophysiological assay would address the question whether reduction in Aβ via inhibition of γ-secretase impacts hippocampal theta oscillation and impairs cognitive function. Furthermore, impact of Aβ reduction on hippocampal theta oscillation by compounds acting via different mechanisms, such as γsecretase modulators, Aβ antibodies, or β-secretase inhibitors would be also informative, as diverse treatments differently change composition of Aβ species and APP cleavage products in the brain. For example, differences in levels of the β-C-terminal fragment (β-CTF) of APP following GSIs and a γ-secretase modulator was considered as a critical factor in their effect on cognition: the GSI semagacestat increased β-CTF level and impaired cognitive function in wild-type mice whereas a second-generation γ-secretase modulator neither increased β-CTF level nor impaired cognition (Mitani et al., 2012). In addition to its role in generation of Aβ from APP, γ-secretase also cleaves >80 discrete substrates, many of them critical components of cell signaling, including Notch, ErbB4, E- and N-cadherins, CD44, CD46, the low-density lipoprotein receptor, Nectin-1, and the Notch ligands Delta and Jagged (Haapasalo and Kovacs, 2011). Whether inhibition of the processing of any of these substrates results in impaired signaling that could lead to the detrimental cognitive effects observed in the semagacestat clinical trial is thus far unknown.

It is also a possibility that reduction in elicited hippocampal theta oscillation is an off-target effect of semagacestat, independent from its Aβ reducing action. Since sub-chronic administration of semagacestat maximally inhibited stimulation-induced theta power without reduction in frequency, the 10-times higher brain concentration of semagacestat following acute, s.c. administration elicited similar effects and did not impact theta frequency either. The short-onset effect of semagacestat following acute administration is similar to those responses which are recorded after application of direct-acting receptor antagonists, such as the muscarinic or NMDA receptor antagonists (Kinney et al., 1999; Siok et al., 2006; Li et al., 2007; McNaughton et al., 2007; Guadagna et al., 2012; Kittelberger et al., 2012; Kiss et al., 2013). The muscarinic receptor antagonist atropine reduces elicited theta oscillation predominantly at lower but not higher frequency range (Li et al., 2007), whereas the NMDA receptor antagonists ketamine attenuates theta power across the whole theta frequency range (Kittelberger et al., 2012) as semagacestat did in the present study. However, receptor-binding studies on semagacestat did not reveal an obvious or probable off-target of semagacestat for this effect (CEREP profile, Pfizer in-house database).

Testing stimulation-induced theta oscillation is considered as a valuable pharmacodynamic assay with high predictive validity of cognitive disruption, but it provides limited information about modulation of physiological hippocampal theta activity. Hippocampal theta oscillation is present during active wakefulness and orientation and rapid eye movement sleep, as well as it plays a role in memory encoding and affective functions in experimental animal (Buzsaki, 2002) and humans (Lega et al., 2012). Therefore, it would be important to test semagacestat if it impacts physiological theta oscillation during particular behaviors and/or cognitive processes, as it could contribute to our understanding its mode of action for worsening AD symptoms in patients.

## Conflict of Interest Statement

All authors either are or have been employees of Pfizer Inc. and own its publicly traded common shares. This work was supported by Pfizer Global Research and Development, Groton, CT, USA.
